# Case Report: Extraskeletal osteosarcoma with preceding myositis ossificans

**DOI:** 10.3389/fonc.2023.1024768

**Published:** 2023-02-24

**Authors:** Hiroki Imada, Tomoaki Torigoe, Yasuo Yazawa, Satoshi Kanno, Jiro Ichikawa, Takehiko Yamaguchi, Tomonori Kawasaki

**Affiliations:** ^1^ Department of Pathology, Saitama Medical University Saitama Medical Center, Kawagoe, Japan; ^2^ Department of Orthopaedic Oncology and Surgery, Saitama Medical University International Medical Center, Hidaka, Japan; ^3^ Department of Pathology, Saitama Medical University International Medical Center, Hidaka, Japan; ^4^ Department of Orthopedic Surgery, Graduate School of Medical Science, University of Yamanashi, Chuo, Japan; ^5^ Department of Pathology, Dokkyo Medical University Nikko Medical Center, Nikko, Japan

**Keywords:** extraskeletal osteosarcoma, myositis ossificans, USP6, high-grade sarcoma, malignant transformation

## Abstract

Extraskeletal osteosarcoma (EO) is a soft tissue sarcoma characterized by the production of bone matrix by neoplastic cells. Benign osteoid in EO, leading to a diagnostic dilemma, is rarely encountered. Herein, for the first time, we present a case with cytogenetically confirmed EO combined with or preceding myositis ossificans (MO). A 21-year-old man had a mildly painful swelling in his left knee. Imaging studies demonstrated a 39-mm mass with peripheral mineralization and cystic change on the posterolateral side of the left fibular head. He was clinically suspected of having either MO or a malignancy, such that wide resection was performed. Macroscopically, the mass was grayish to brown. In the cut section, multiple cystic lesions in addition to solid components were noted. Histopathologically, the solid components demonstrated diffuse proliferation of pleomorphic tumor cells with osteoclast-like giant cells. The malignant tumor cells formed osteoid. In the periphery, the mass was benign, showing mature bone tissue and focally non-malignant woven bone with fibroblasts, compatible with zonation. Fluorescence *in situ* hybridization (FISH) demonstrated split signals of the *USP6* gene. These findings suggested EO with preceding MO. Although the pathogenesis remains to be elucidated, the observed *USP6* rearrangement might contribute to both the diagnosis of EO with preceding MO and an understanding of the underlying histopathology.

## Introduction

Extraskeletal osteosarcoma (EO) is defined as soft tissue sarcoma with bone matrix production by malignant cells outside the skeletal system (1). The pathological diagnosis of EO is based solely on the identification of histological osteogenic differentiation. The presence of benign osteoid-simulating myositis ossificans (MO) is rarely encountered in EO cases, possibly presenting both diagnostic and etiologic dilemmas. EO has been recognized as having complex genomic signatures (2), but most of the genetic alterations discovered to date have been non-diagnostic in clinical practice. On the other hand, MO was recently found to harbor a *USP6* rearrangement (3). However, unfortunately, the previous case reports of EO with MO lacked cytogenetic investigation. To our knowledge, this is the first report to describe a cytogenetically confirmed case in which EO was combined with or preceded MO. This is also only the second description of EO with distinct zonation of MO.

## Clinical summary

**Figure 1:**
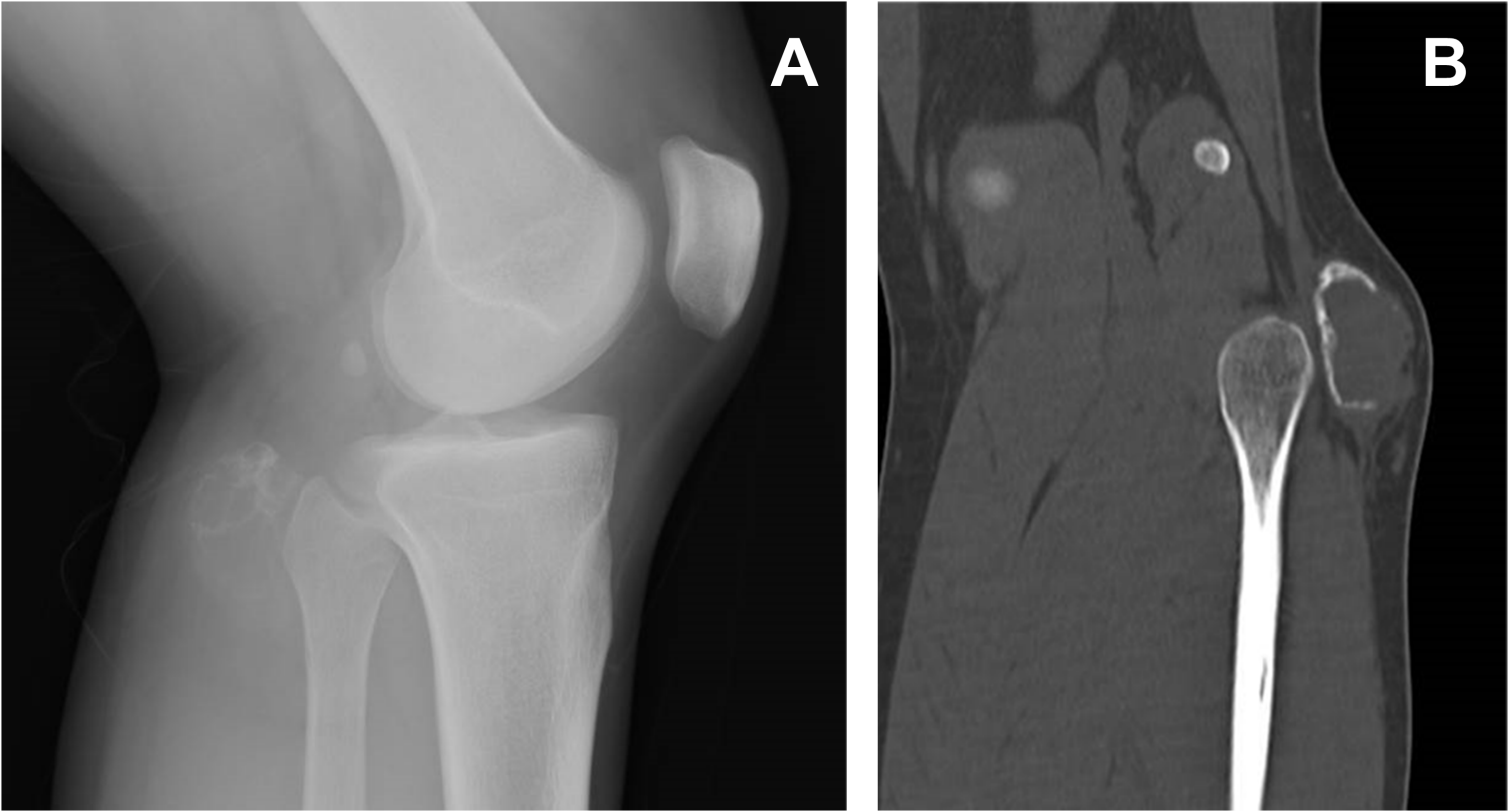
Lateral view x-ray of the left knee demonstrating a mass posterior to the fibular head **(A)**. On reconstructed CT, the mass was calcified in the periphery, but the shell was not complete and showed focal interruption **(B)**.

**Figure 2:**
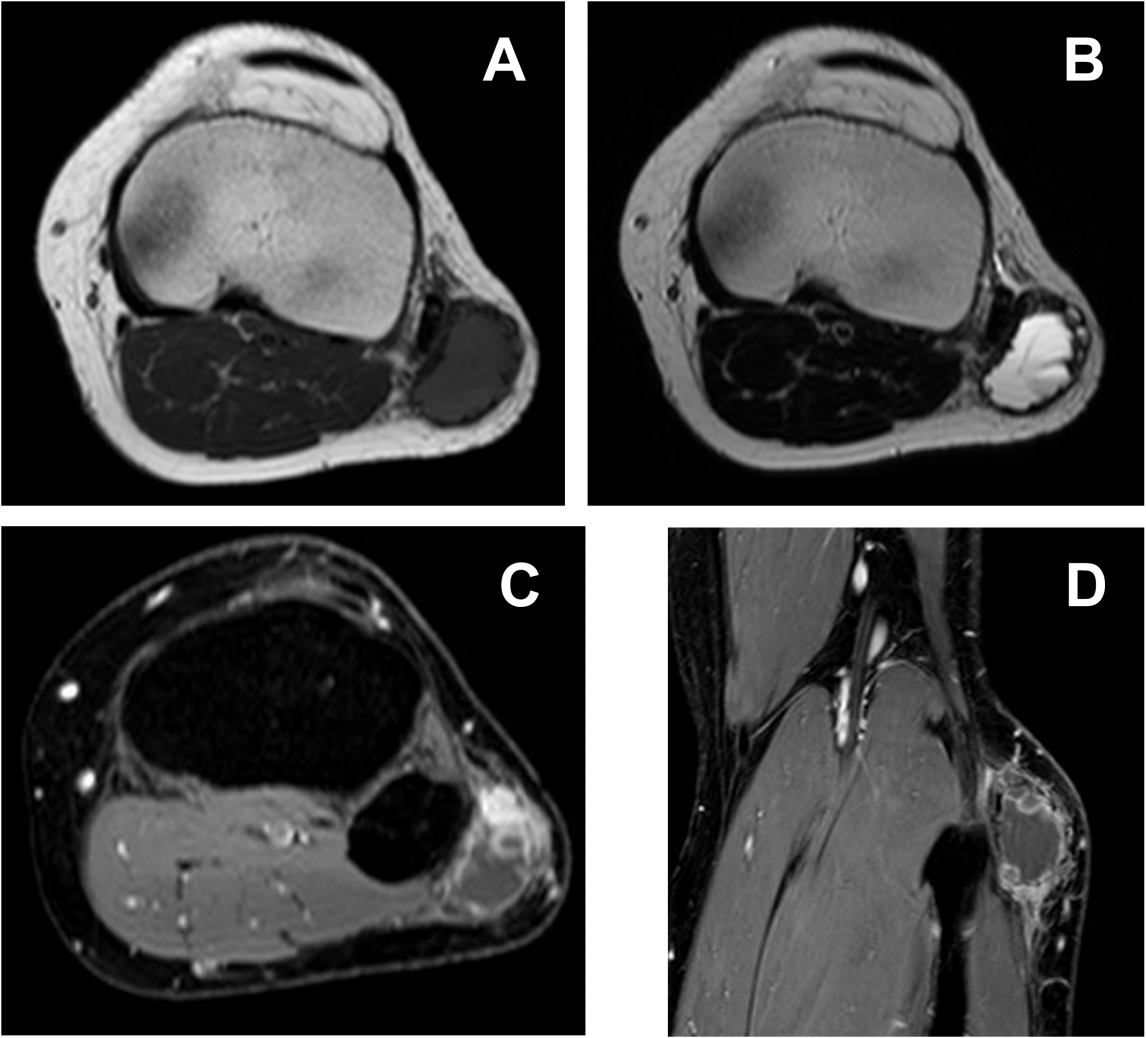
On T1-weighted imaging **(A)**, the mass shows intermediate signal intensity, while heterogeneously high intensity with fluid–fluid level can be seen on T2-weighted imaging **(B)**. Peripheral and nodular enhancement is recognizable on FS-T1-weighted imaging with contrast **(C, D)**.

A 21-year-old man consulted our hospital for a mildly painful swelling on his left knee that he had noticed 4 days earlier. A 40 × 40 × 30 mm subcutaneous mass was identified on the lateral side of the left fibular head. Laboratory test results were unremarkable. X-rays and computed tomography (CT) scans revealed a mass lesion on the posterolateral side of the left knee and the periphery of the mass showed mineralization (**Figure 1**). There was no continuity with the bone cortex. On magnetic resonance imaging (MRI), the mass was solid and cystic with intermediate signal intensity on T1-weighted imaging (**Figure 2A**), while showing heterogeneously high intensity with the fluid–fluid level on T2-weighted imaging (**Figure 2B**). The periphery was enhanced on FS-T1-weighted imaging with contrast (**Figures 2C, D**). His prior medical history, except for an injury to the same knee 10 years earlier, was unremarkable and he had never undergone radiation therapy. No evidence of metastasis was identified clinically. The core needle biopsy specimen was insufficient and did not yield a diagnosis. However, the cytology specimen suggested malignancy. Due to the tendency of the lesion to show growth and the positive cytology result, the tumor was clinically suspected of MO or hematoma with malignancy. The mass lesion was thus excised with a wide margin containing biopsy tract, without preoperative chemotherapy 6 weeks after the initial presentation. At the time of excision, the mass had not changed in size since the initial visit.

## Pathological findings

**Figure 3:**
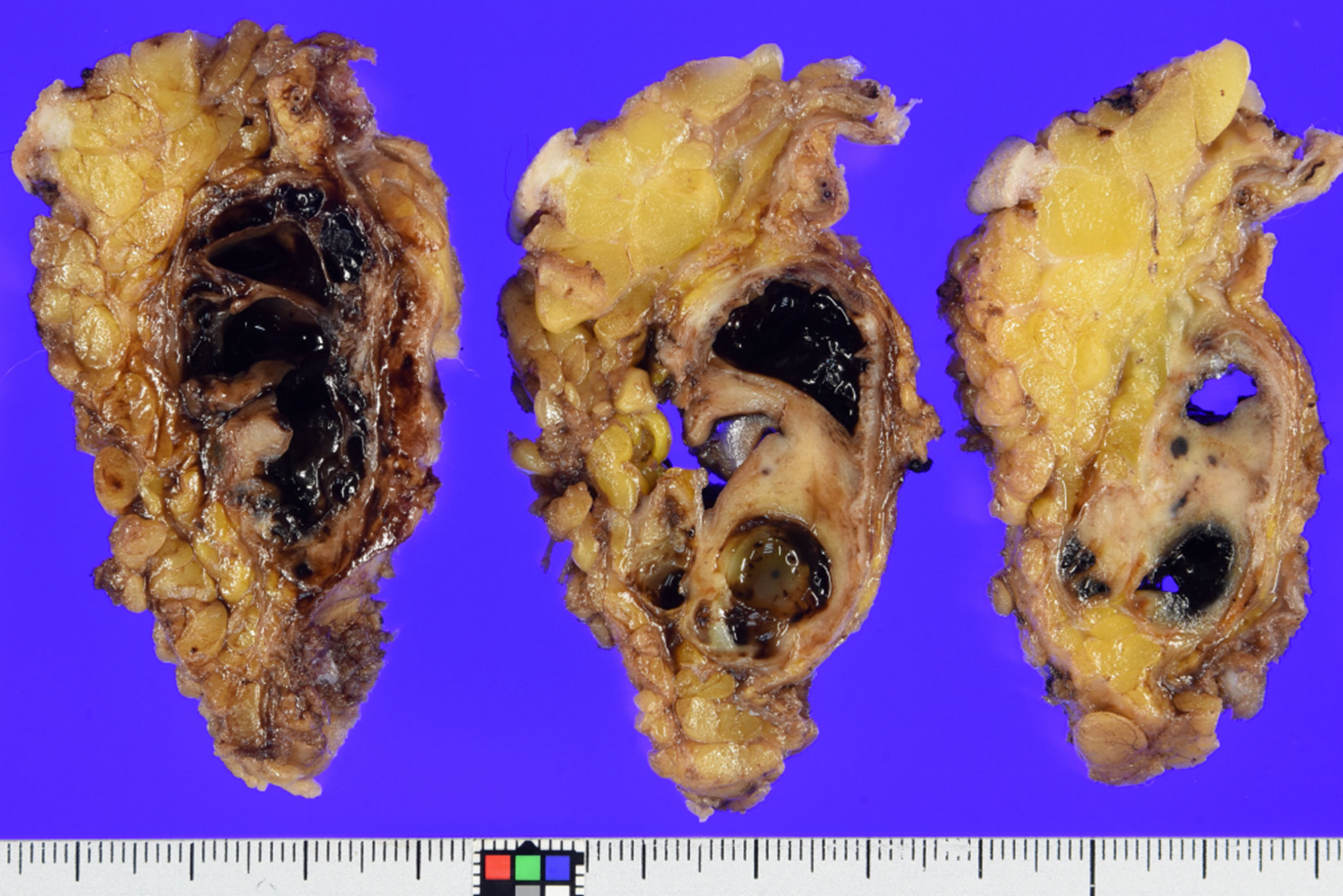
Grossly, on the cut section, the multicystic mass is seen to be subcutaneously located and mixed with hemorrhagic change and solid components. Mineralization was confirmed in the periphery, which corresponded to the imaging findings.

**Figure 4:**
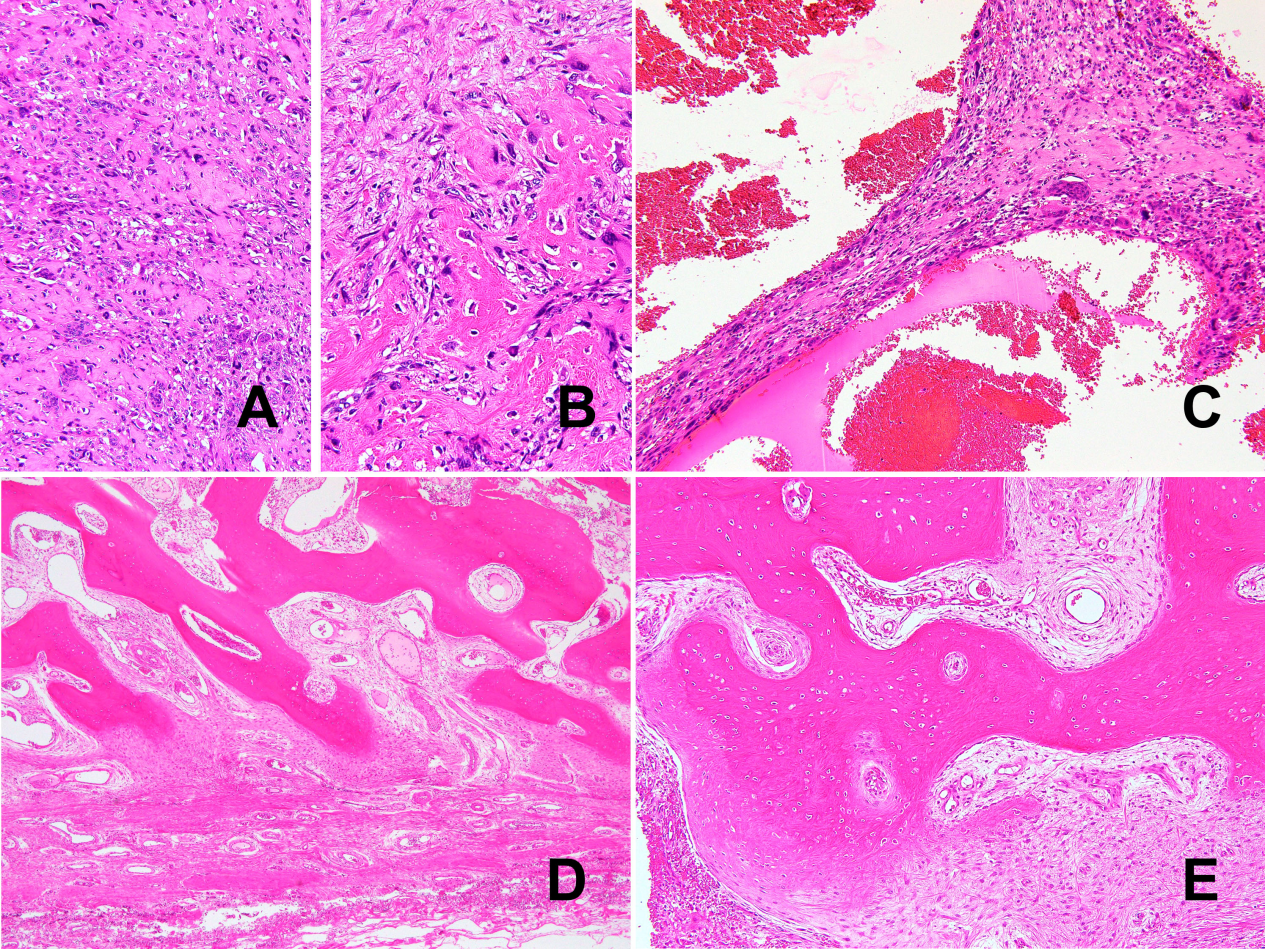
Histologically, the tumor was a high-grade pleomorphic sarcoma **(A)** with osteoid formation **(B)**. Telangiectatic or aneurysmal bone cyst-like change with abundant osteoclast-like giant cells can also be seen **(C)**. Morphologically bland-appearing, mature bone area in the periphery of the mass. **(D)**. In addition to mature lamellar bone, woven bone and fibroblasts show a continuous pattern, consistent with zonation **(E)**.

Macroscopically, the gray-whitish or blackish-brown mass was located in the subcutaneous tissue, measuring 4.5 × 4 × 2.5 cm. On the cut surface, multiple cystic lesions filled with blood were noted with focal solid components (**Figure 3**). Histologically, the solid components were composed of diffuse proliferation of pleomorphic tumor cells, intermingled with abundant osteoclast-like giant cells (**Figure 4A**). Mitotic figures were numerous [22 per 10 high-power fields (HPFs)], with atypical mitoses. The tumor cells focally formed neoplastic osteoid (**Figure 4B**). No other morphological differentiation was confirmed. Cystic lesions included bloody materials and, internally, osteoclast-like giant cells (**Figure 4C**), compatible with telangiectatic or aneurysmal bone cyst-like change. The periphery of the mass consisted of benign mature bone with bland osteocytes (**Figure 4D**). The mature bone area was predominantly composed of lamellar bone trabeculae and focally woven bone with fibroblasts, and thus zonation was suggested (**Figure 4E**). EO components were adjacent to but discontinuous with mature bone lesions.

**Figure 5:**
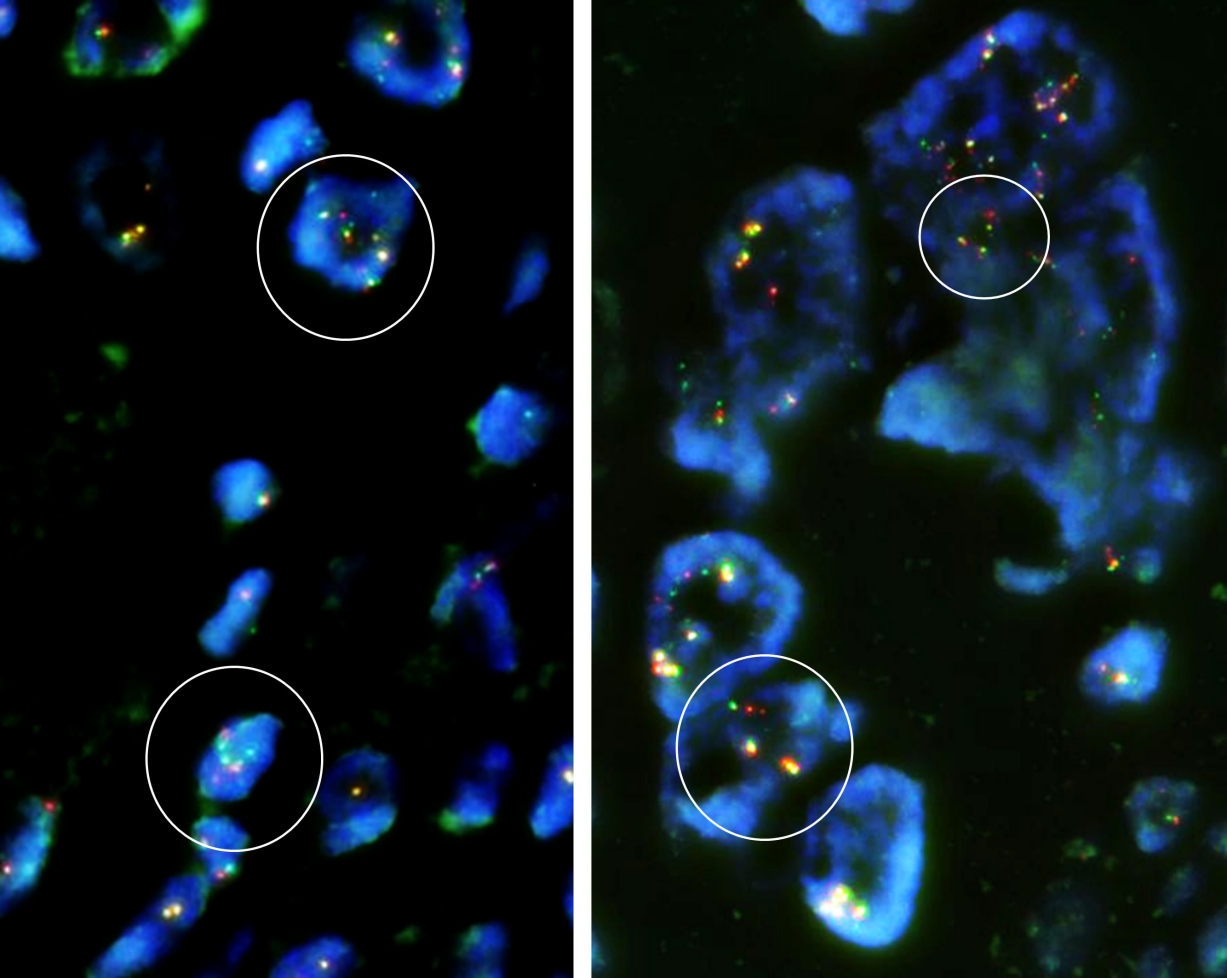
Fluorescence *in situ* hybridization (FISH) demonstrated break-apart signals of the *USP6* gene (*USP6* Split Dual Color FISH Probe, GSP Lab) in the EO component. Some of the tumor cells are polyploid, and focally split signals of the *USP6* gene are identifiable in the tumor cells (circled in white), compatible with rearrangement of the *USP6* gene.

Immunohistochemically, the tumor cells showed no expression of MDM2 (Clone IF2, dilution 100×, ThermoFisher), CDK4 (Clone DCS-31, dilution 200×, Invitrogen), or H3.3 G34W (Clone RM263, dilution 800×, RevMAb Biosciences). Fluorescence *in situ* hybridization (FISH) revealed split signals of the *USP6* gene in the tumor cells (**Figure 5**). Based on these pathological findings, we diagnosed EO with the preceding MO.

The patient remains alive and limb function is completely preserved. Neither recurrence nor metastasis was recognized 16 months after surgery.

## Discussion

MO has been regarded as an aggressively reactive process with bone formation (4). It commonly occurs in athletically active young men often with a prior history of trauma or surgery, and patients usually present with a painful mass (4). The precise etiology of MO has yet to be elucidated, but an inappropriate metaplastic change or osteogenic differentiation of fibroblasts has been proposed (4, 5). The presence of a zonal pattern of bone trabeculae is considered to be a distinctive feature of MO (4), but the zonation is ill-defined in some cases, especially in those with long-standing lesions. Rearrangement of the *USP6* gene was recently reported in MO (3). MO has also been recategorized as one of the *USP6*-associated neoplasms (UAN), along with other tumorous lesions showing *USP6* rearrangement, which suggests neoplastic change rather than a reactive process (6).

On the other hand, EO is a high-grade sarcoma with osteoid or bone matrix production by malignant neoplastic cells, and, by definition, the tumor has no connection to the skeletal system (1). EO is rare, comprising less than 1% of all soft tissue sarcomas and only about 4% of osteosarcomas overall (1, 7). The lower extremities, especially the thighs, are the most common site of involvement (1). Middle-aged to older patients, with a slight male predominance, tend to be affected (1). EO can show various histological subtypes as with conventional osteosarcoma, and telangiectatic or aneurysmal bone cyst-like change has been reported (8). EO is usually diagnosed based on the detection of malignant osteoid formation and by ruling out other sarcomas with osteogenic differentiation such as dedifferentiated liposarcoma. Although the exact etiology of EO has not been clarified, the genomic signature has revealed features overlapping those of conventional skeletal osteosarcoma (1, 2). Because EO is usually high-grade, mature bone is not a typical feature and can, therefore, pose a diagnostic dilemma as in our present case.

At the center of the tumor in this patient, there was a high-grade pleomorphic sarcoma with malignant osteoid formation and telangiectatic change. This telangiectatic change corresponded to the fluid–fluid level on MRI (**Figure 2**). In the periphery of the tumor, mature bone trabeculae surrounded the high-grade osteosarcoma component, which was recognized on x-ray and CT scans (**Figure 1**). Focal zonation was confirmed in the peripheral benign bone area.

Differential diagnoses in our case included ossifying fibromyxoid tumor (OFMT) (9), osteosarcoma arising from the tiny bone or ectopic/metaplastic bone tissue (4, 9), and EO with preceding MO. OFMT is characterized by the proliferation of uniform round cells with peripheral ossification and is characterized by a rearrangement of the *PHF1* gene. The mass in our case was different from OFMT in that the tumor cells were pleomorphic. We were not able to identify either ectopic bone or continuity with the adjacent bone cortex. Therefore, OFMT and tiny bone osteosarcoma were ruled out, based on the factors discussed above. We confirmed the *USP6* rearrangement in the tumor cells, a finding not previously reported in EO. Based on the *USP6* rearrangement and peripheral mature bone tissue with focal zonation, we definitively diagnosed EO with preceding MO.

Malignant transformation of MO or osteosarcoma arising from preexisting MO is exceedingly rare, but several cases have been reported to date (4, 5, 7, 9). In these past reported cases, the ages ranged from 19 to 53 (mean, 37) years, and there was no gender predilection (4). The thigh was the location most commonly involved, though other areas including the jaw, retroperitoneum, wrist, and knee have also been reported (4). Benign bone tissue or MO component is commonly recognized in the periphery of the mass (7, 9). The prognosis is controversial but outcomes are generally dismal with most of the reported patients dying within 6 years of the initial presentation (4). The EO component is usually high-grade, but in one case report, a low-grade osteosarcoma with MO was described (10).

Most prior cases presented a diagnostic dilemma because they were diagnosed based on radiological and/or histological evidence of heterotopic benign bone tissue associated with EO (7, 9). The characteristic zonal distribution was confirmed in only one case (4). Furthermore, cytogenetic findings were not investigated in the previous cases, probably because osteosarcoma was long assumed to harbor no diagnostic cytogenetic abnormalities (1). As MO was known to harbor the *USP6* rearrangement, we speculated that *USP6* rearrangement could be detected in the sarcoma component and thus be a key factor in identifying EO arising from MO (3).

While there is no established criterion for diagnosing EO arising from MO, previously reported cases had difficulty proving the typical MO component and some of these cases are thus diagnostically questionable. In our current case, however, we identified a distinct zonation of the peripheral bone tissue that was etiologically significant in MO, as well as the *USP6* rearrangement in the high-grade osteosarcoma component that existed in the center of the tumor. Our case is the first to provide confirmation of the rearrangement of *USP6* and the second in which the zonation of the MO component was identified. These observations support the diagnosis of EO arising from MO and suggest that MO is a neoplastic process. Unusual *USP6* rearrangement in EO may also be interpreted as MO with malignant transformation.

We have presented an exceptionally rare case of EO with preceding MO, and *USP6* rearrangement appears to reinforce the concept of malignant transformation of MO. Although the precise etiology of EO accompanied by MO remains as yet poorly understood, our current case provides the first confirmation of the rearrangement of *USP6* and is the second demonstration of zonation of the MO component.

## Data availability statement

The original contributions presented in the study are included in the article/supplementary materials. Further inquiries can be directed to the corresponding author.

## Ethics statement

Ethical approval was not provided for this study on human participants because Our hospital does not require ethical approval in case reports. The patients/participants provided their written informed consent to participate in this study.

## Author contributions

Conception and design of the study: HI and TK. Acquisition and analysis of data: HI, TK, TT, YY and SK. Drafting the manuscript or figures: HI, TK, JI and TY. All authors contributed to the article and approved the submitted version.
